# Trends and inequalities in health facility deliveries among women of reproductive age in Ghana, 1993–2022

**DOI:** 10.3389/frph.2026.1874042

**Published:** 2026-07-03

**Authors:** Patience Fakornam Doe, Frank Offei Odonkor, Yvonne Dorothy Mintah, Joseph Lasong, Yula Salifu, Amidu Alhassan

**Affiliations:** 1 Department of Public Health, School of Nursing and Midwifery, College of Health and Allied Sciences, University of Cape Coast, Cape Coast, Ghana; 2 Department of Adult Health, School of Nursing and Midwifery, College of Health and Allied Sciences, University of Cape Coast, Cape Coast, Ghana; 3 Department of Population and Reproductive Health, School of Public Health, University for Development Studies, Tamale, Ghana; 4 Department of Population, Family and Reproductive Health, School of Public Health, University of Ghana, Accra, Ghana; 5 Department of Nursing, School of Biomedical Engineering and Allied Health Sciences, All Nations University, Koforidua, Ghana

**Keywords:** Ghana, health facility deliveries, inequalities, maternal health, socioeconomic disparities

## Abstract

**Background:**

Health facility delivery is a critical intervention to reduce maternal and neonatal mortality. Ghana has achieved substantial gains in facility deliveries over the past three decades, yet disparities persist across socioeconomic and demographic dimensions. This study describes trends and inequalities in health facility deliveries among women of reproductive age in Ghana from 1993 to 2022 across wealth, education, place of residence, age, and region.

**Methods:**

Data were extracted from the WHO Health Equity Assessment Toolkit (HEAT), which provides weighted, age-standardised estimates from Ghana Demographic and Health Surveys (DHS) and Multiple Indicator Cluster Surveys (MICS) for the years 1993, 1998, 2003, 2006, 2008, 2011, 2014, 2017, and 2022. The outcome was the percentage of live births occurring in a health facility (WHO HEAT indicator code: MNCH7). Inequality was measured using the absolute difference (D), ratio (R), population attributable fraction (PAF), and population attributable risk (PAR) for each dimension, with 95% confidence intervals.

**Results:**

National health facility delivery prevalence increased from 42.3% (95% CI: 39.5–45.1) in 1993 to 85.4% (95% CI: 84.1–86.7) in 2022. In 2022, marked disparities remained: richest vs. poorest quintile (97.1% vs. 71.1%; D = 25.7 percentage points); higher education vs. no education (98.0% vs. 72.3%; D = 25.7 percentage points); urban vs. rural (93.7% vs. 77.9%; D = 15.8 percentage points); and Upper East vs. Northern region (97.7% vs. 69.0%; D = 28.7 percentage points). Absolute economic inequality (D) declined from 60.6 percentage points in 1993 to 25.7 in 2022; educational inequality fell from 53.6 to 17.8; and rural–urban inequality declined from 35.8 to 9.7. Age-related inequality was negligible throughout.

**Conclusion:**

While Ghana has achieved substantial gains in health facility deliveries, marked inequalities by wealth, education, place of residence, and region persist. These patterns coincide temporally with policy reforms including the Free Maternal Health Policy and the National Health Insurance Scheme; however, this descriptive analysis cannot establish causality. Targeted interventions for women in poorer, less educated, and rural settings are needed to achieve equitable maternal healthcare.

## Introduction

The provision of skilled care during childbirth is a critical determinant of maternal and neonatal health outcomes, with health facility delivery playing a central role in ensuring that women and infants receive necessary medical care in the event of complications. The World Health Organization (WHO) recommends that all births be attended by skilled healthcare professionals, as this significantly reduces maternal and neonatal mortality (1). In Ghana, health facility delivery has improved substantially over the past three decades, yet disparities remain, particularly between urban and rural regions (2).

Globally, the importance of skilled birth attendance is widely acknowledged, with an increase from 20% points from 64% on average during 2001–2007 to 84% during 2015–2022 (3). In sub-Saharan Africa, the figure is lower, at 85%, but countries like Rwanda and Kenya have seen notable improvements (4). In Ghana, the prevalence of health facility deliveries has risen from 42% to 86% (5). However, these national improvements conceal significant regional variations, with urban areas experiencing higher rates of health facility deliveries compared to rural regions. For example, Greater Accra and Ashanti Regions have consistently reported higher rates of facility-based deliveries, while rural regions, including Northern and Volta, have lagged (6). This disparity indicates that while national progress is encouraging, subnational inequalities persist and require closer examination.

The factors contributing to these disparities are multifaceted and include place of residence, economic status (7), age, and education level (8, 9). Place of residence remains a key determinant of access to health facilities, with rural areas often facing challenges such as long distances to healthcare facilities (10, 11), poor road infrastructure (12), and limited availability of skilled healthcare providers (13). Economic status also plays a critical role, as individuals from lower-income households may be unable to afford transportation to health facilities or face financial barriers related to healthcare services, such as fees for delivery care (14). Age is another significant factor, with younger women, particularly those under 20 years, less likely to seek institutional delivery due to limited knowledge, cultural norms, or lack of support (15, 16). Lastly, education level is closely linked to health-seeking behaviours, as educated women are more likely to understand the benefits of skilled attendance during childbirth and are better equipped to navigate healthcare systems (4, 17, 18). These factors, individually and collectively, continue to influence health facility delivery rates and contribute to ongoing regional inequalities.

While Ghana has made significant strides in increasing the proportion of deliveries occurring in health facilities, regional disparities persist, and inequalities based on place of residence, economic status, age, and education continue to affect maternal health outcomes. Although the national prevalence of health facility deliveries has increased, these improvements are not equitably distributed across the country. Rural areas, lower-income households, younger women, and those with lower levels of education face significant barriers to accessing skilled care during childbirth (19–22). Existing studies have primarily focused on national trends (23–25), with limited attention to how these key socio-economic and demographic factors influence health facility delivery rates at the subnational level. This gap in knowledge hinders the development of targeted interventions that can address the unique needs of different populations, leading to continued inequities in maternal and child health outcomes across Ghana. This study aims to examine the progress and persistent inequalities in the prevalence of deliveries in health facilities among women of reproductive age in Ghana from 1993 to 2022, with a focus on how factors such as place of residence, economic status, age, and education contribute to these disparities. By analysing disaggregated data, the research seeks to identify the underlying barriers and facilitators to health facility delivery at the regional level. The findings will provide valuable insights into how these socio-economic and demographic factors continue to influence access to skilled care during childbirth in Ghana. Ultimately, the study will contribute to the development of region-specific interventions and policies aimed at improving health facility delivery rates and reducing maternal and neonatal mortality in underserved regions of the country.

## Methods

### Data source and study design

This study is a secondary data analysis using a descriptive, cross-sectional repeated-survey design. Data were extracted from the WHO Health Equity Assessment Toolkit (HEAT): a software application that provides pre-calculated, age-standardised health inequality estimates drawn from nationally representative household surveys. For Ghana, HEAT draws on the Demographic and Health Surveys (DHS) and the Multiple Indicator Cluster Surveys (MICS). We extracted all available survey rounds that included the indicator “deliveries in a health facility” (WHO HEAT indicator code: MNCH7). The survey years included were: 1993, 1998, 2003, 2006, 2008, 2011, 2014, 2017, and 2022.

The denominator population for each estimate comprises all live births in the five years preceding the survey (the standard DHS/MICS definition). All estimates are weighted using survey-specific sampling weights to adjust for unequal probability of selection and non-response, thereby ensuring national representativeness. The unweighted sample sizes (number of women with at least one live birth in the five-year recall period) were: 1993: n = 4,562; 1998: n = 4,843; 2003: n = 5,691; 2006: n = 4,205; 2008: n = 3,896; 2011: n = 5,816; 2014: n = 5,038; 2017: n = 6,921; 2022: n = 7,845. Missing data on place of delivery were consistently below 1% across all surveys; HEAT implements complete-case analysis for indicator calculation.

### Outcome measure

The primary outcome is the prevalence of deliveries in a health facility, defined as the percentage of live births that occurred in a health facility (hospital, health centre, clinic, or maternity home). This indicator is extracted directly from WHO HEAT (indicator code MNCH7) and is based on the survey question: “Where did you give birth to [name]?”. Skilled birth attendance is not used as an outcome; all analyses are confined exclusively to facility-based deliveries.

### Dimensions of inequality

Disparities in health facility delivery prevalence were examined across five dimensions of inequality, as defined by WHO HEAT: (1) age (women aged 15–19 years vs. 20–49 years); (2) economic status (wealth quintiles — poorest, poorer, middle, richer, richest: derived from DHS/MICS asset-based indices); (3) education (no education, primary, secondary, higher); (4) place of residence (urban vs. rural); and (5) subnational region (all administrative regions of Ghana, harmonised by WHO HEAT to consistent units over time).

### Statistical analyses

All analyses were performed using the built-in functions of WHO HEAT (version 3.1). For each survey year, point estimates (percentages) with 95% confidence intervals (CI) were calculated for health facility delivery prevalence at the national level and for each subgroup. CIs for simple proportions were computed using the exact binomial (Clopper–Pearson) method.

To quantify and compare inequalities over time, four summary measures were used following standard WHO HEAT methodology:

**Difference (D):** An absolute measure of inequality calculated as the difference between the most advantaged (e.g., richest quintile or highest education) and most disadvantaged subgroup (e.g., poorest quintile or no education). D is expressed in percentage points. Larger positive values indicate greater absolute inequality favouring the advantaged group. Formula: $D=P_{richest}-P_{\;poorest}$ (for economic status).
**Ratio (R):** A relative measure calculated as the prevalence in the most advantaged subgroup divided by that in the most disadvantaged subgroup. R > 1 indicates higher prevalence in the advantaged group. Formula: $R=P_{richest}/P_{\;poorest}$.
**Population Attributable Fraction (PAF):** Measures the proportional reduction in national prevalence that would result if the disadvantaged subgroup had the same prevalence as the reference subgroup, expressed as a percentage. Formula: $PAF=\frac{P_{national}-P_{disadvantaged}}{P_{national}}\times 100\text{\%}$.
**Population Attributable Risk (PAR):** The absolute version of PAF, expressing in percentage points how much the national prevalence exceeds that of the disadvantaged subgroup. Formula: $PAR=P_{national}-P_{disadvantaged}$.

Standard errors and 95% CIs for D and R were calculated using the analytic (delta) method implemented in HEAT. For PAF and PAR, CIs were derived via Monte Carlo simulation (1,000 replications) as recommended by WHO. All inequality measures were calculated separately for each survey year to allow comparison of trends over time. Results are presented in tables and figures with point estimates and 95% CIs.

## Results

### National prevalence of health facility deliveries among women of reproductive age in Ghana

**Figure 1:**
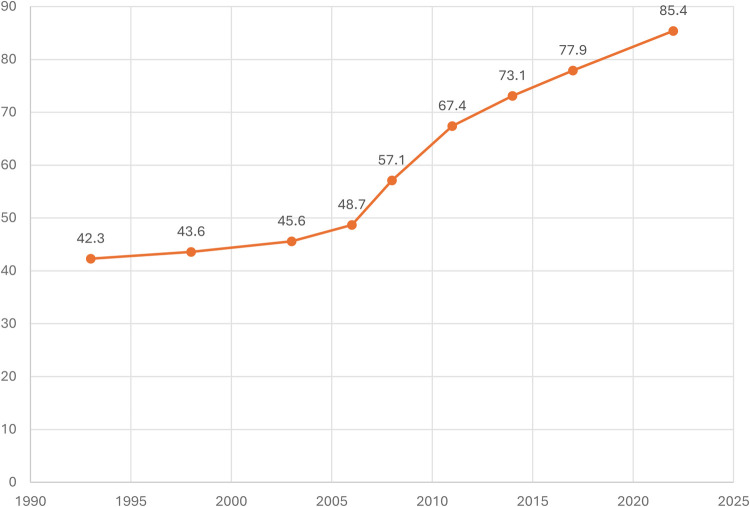
National prevalence of health facility deliveries among women of reproductive age in Ghana, 1993–2022. Source: WHO HEAT; indicator MNCH7; Ghana DHS/MICS survey rounds.

Figure 1 presents the national prevalence of health facility deliveries among women of reproductive age in Ghana from 1993 to 2022. Prevalence increased from 42.3% (95% CI: 39.5–45.1) in 1993 to 45.6% (42.6–48.7) in 2003, 57.1% (53.9–60.2) in 2008, 73.1% (70.5–75.6) in 2014, and 85.4% (84.1–86.7) in 2022, representing an absolute increase of 43.1 percentage points over the study period.

### Regional distribution of health facility deliveries among women of reproductive age in Ghana

**Figure 2:**
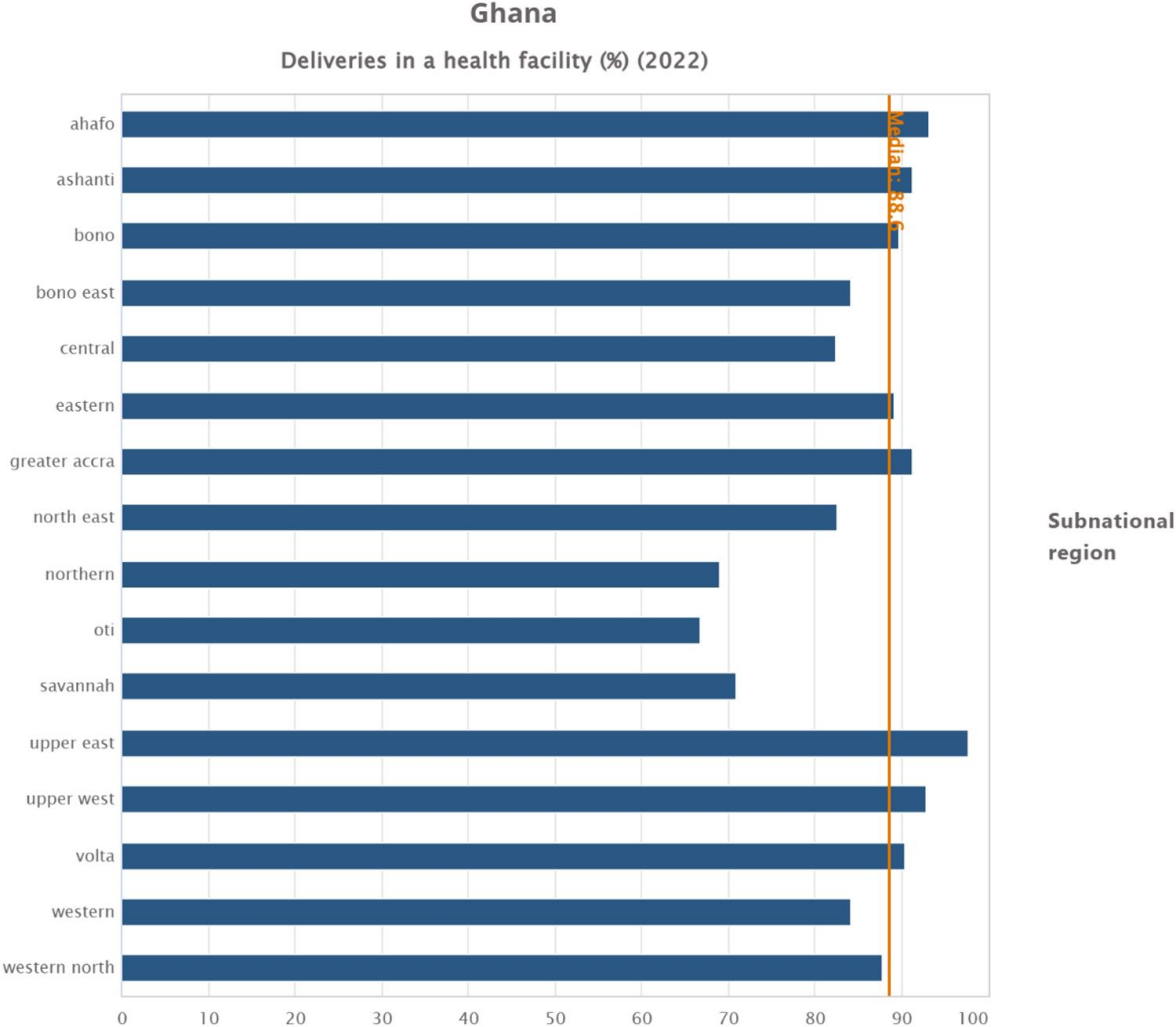
Regional distribution of health facility delivery prevalence in Ghana, 2022. Source: WHO HEAT; indicator MNCH7; Ghana DHS 2022.

Figure 2 presents the regional distribution of health facility delivery prevalence in 2022. The highest prevalence was observed in the Upper East region (97.7%, 95% CI: 95.4–98.9), followed by Upper West (92.8%, 88.4–95.7), Greater Accra (91.3%, 86.8–94.4), and Ashanti (91.3%, 87.0–94.3). The lowest prevalence was recorded in the Northern region (69.0%, 95% CI: 57.0–78.9), representing a regional gap of 28.7 percentage points between the highest- and lowest-performing regions in 2022.

### Trends in health facility deliveries among women of reproductive age by education and place of residence

**Figure 3:**
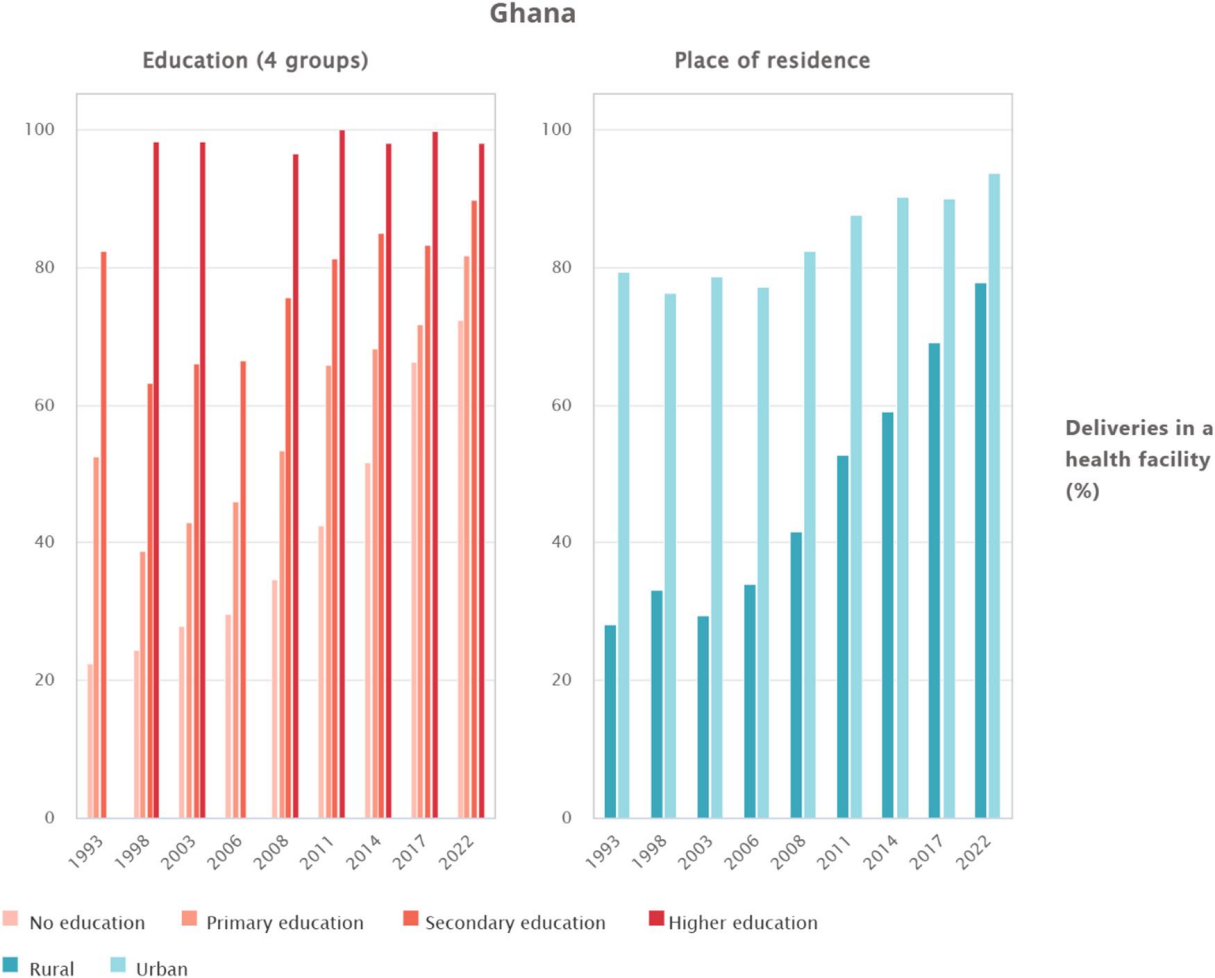
Trends in health facility delivery prevalence among women of reproductive age in Ghana by education and place of residence, 1993–2022. Source: WHO HEAT; indicator MNCH7; Ghana DHS/MICS survey rounds.

Figure 3 presents trends in health facility delivery prevalence by education and place of residence from 1993 to 2022. Women with higher education consistently recorded the highest rates, rising from approximately 60% in 1993 to 98.0% (95% CI: 95.2–99.2) in 2022. Women with secondary education increased from approximately 50% in 1993 to 89.7% (95% CI: 87.8–91.4) in 2022. Women with no education recorded the lowest rates, rising from 22.4% (95% CI: 19.0–26.2) in 1993 to 72.3% (95% CI: 66.4–77.5) in 2022. Women with primary education saw a steady rise over the same period, reaching approximately 75%–78% by 2022.

In terms of place of residence, urban women consistently had higher delivery rates in health facilities, reaching 93.7% (95% CI: 92.2–94.9) in 2022, compared with 77.9% (95% CI: 74.2–81.1) in rural areas. Rural women showed considerable improvement from 28.0% (95% CI: 24.4–31.9) in 1993, though rural populations continue to remain behind their urban counterparts.

### Trends in health facility deliveries among women of reproductive age by age and economic Status

**Figure 4:**
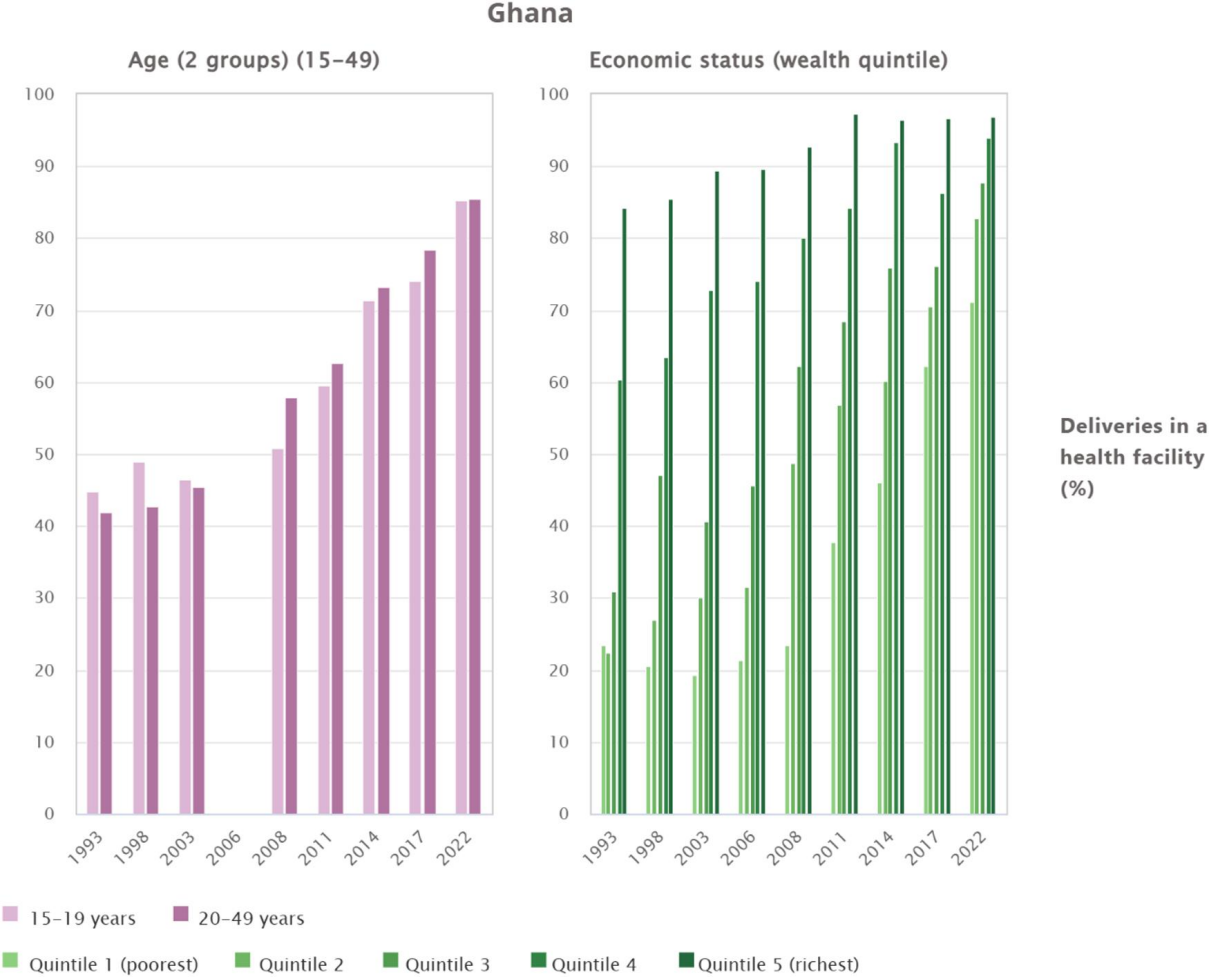
Trends in health facility delivery prevalence among women of reproductive age in Ghana by age group and economic status, 1993–2022. Source: WHO HEAT; indicator MNCH7; Ghana DHS/MICS survey rounds.

Figure 4 depicts trends in health facility delivery prevalence by age group and economic status from 1993 to 2022. Among women aged 15–19, prevalence rose from 44.9% (95% CI: 38.5–51.5) in 1993 to 85.3% (95% CI: 81.2–88.6) in 2022, while women aged 20–49 increased from 41.9% (95% CI: 38.6–45.3) to 85.4% (95% CI: 83.3–87.4) over the same period. Age-related inequality was negligible throughout (D < 0.3 percentage points in all survey years).

Economic status showed a more pronounced disparity over the study period. In 1993, only 23.5% (95% CI: 18.3–29.7) of women in the poorest quintile delivered in a health facility, compared with 70.4% (95% CI: 64.2–76.0) of those in the richest quintile. By 2022, coverage among the poorest quintile reached 71.1% (95% CI: 64.8–76.7), while the richest quintile approached near-universal coverage at 97.1% (95% CI: 95.7–98.1). The steepest gains among the poorest occurred between 2003 and 2014.

### Summary measures of inequality in health facility deliveries among women of reproductive age, 1993–2022

Table 1 presents summary inequality measures for all five dimensions across selected survey years. Economic inequality, measured by the absolute difference (D), fell from 60.6 percentage points (95% CI: 53.5–67.7) in 1993 to 25.7 (95% CI: 19.6–31.9) in 2022. The corresponding ratio (R) declined from 3.58 (2.80–4.57) to 1.36 (1.25–1.48). The PAF fell from 98.96% to 13.30%, indicating a marked reduction in the proportional contribution of wealth-related inequality to the national prevalence.

**Table 1:** Summary measures of inequality in health facility deliveries among women of reproductive age in Ghana by dimension, selected survey years (1993–2022).

Dimension	Measure	1993 Est. (95% CI)	2003 Est. (95% CI)	2011 Est. (95% CI)	2017 Est. (95% CI)	2022 Est. (95% CI)
Economic status (wealth quintile)	D	60.6 (53.5–67.7)	70.1 (65.2–75.0)	59.7 (54.8–64.5)	34.5 (28.2–40.9)	25.7 (19.6–31.9)
	R	3.58 (2.80–4.57)	4.61 (3.83–5.57)	2.58 (2.30–2.90)	1.56 (1.41–1.72)	1.36 (1.25–1.48)
	PAF (%)	98.96 (98.85–99.07)	95.95 (95.87–96.03)	44.59 (44.54–44.63)	24.13 (24.11–24.16)	13.30 (13.29–13.32)
	PAR	41.9 (37.2–46.5)	43.8 (40.2–47.4)	30.0 (27.2–32.9)	18.8 (16.8–20.8)	11.4 (9.9–12.8)
Age (15–49 years)	D	0.2 (−4.3–4.6)	0.3 (−4.2–4.8)	0.1 (−4.2–4.5)	0.2 (−4.2–4.6)	0.2 (−4.1–4.5)
	R	1.0 (0.9–1.1)	1.0 (0.9–1.1)	1.0 (0.9–1.1)	1.0 (0.9–1.1)	1.0 (0.9–1.1)
	PAF (%)	0.3 (−6.0–6.5)	0.4 (−6.2–7.0)	0.2 (−6.0–6.4)	0.2 (−6.1–6.5)	0.3 (−5.9–6.4)
	PAR	0.1 (−2.1–2.2)	0.1 (−2.1–2.3)	0.1 (−2.1–2.2)	0.1 (−2.1–2.2)	0.1 (−2.0–2.1)
Education (4 groups)	D	53.6 (47.2–59.9)	58.7 (53.9–63.5)	46.2 (41.7–50.7)	28.7 (23.6–33.8)	17.8 (13.7–21.9)
	R	3.2 (2.6–4.0)	3.5 (2.9–4.2)	2.0 (1.7–2.3)	1.3 (1.1–1.5)	1.1 (0.9–1.3)
	PAF (%)	89.2 (88.7–89.8)	80.9 (80.4–81.4)	41.6 (41.3–41.9)	21.9 (21.7–22.2)	12.5 (12.3–12.7)
	PAR	28.3 (25.6–31.0)	26.7 (24.6–28.8)	14.2 (12.8–15.7)	6.9 (5.6–8.3)	3.3 (2.5–4.2)
Place of residence (urban/rural)	D	35.8 (30.5–41.0)	39.5 (35.1–43.9)	28.4 (24.4–32.4)	16.9 (13.8–20.0)	9.7 (7.2–12.2)
	R	2.1 (1.7–2.6)	2.5 (2.0–3.0)	1.6 (1.3–1.9)	1.2 (1.0–1.5)	1.1 (0.9–1.3)
	PAF (%)	65.0 (64.1–65.9)	54.1 (53.3–54.9)	23.7 (23.2–24.2)	11.2 (10.9–11.5)	6.1 (5.9–6.3)
	PAR	23.6 (20.6–26.6)	21.6 (19.1–24.1)	9.6 (8.0–11.3)	4.0 (2.9–5.2)	1.8 (1.1–2.6)
Subnational region	D	28.1 (24.0–32.1)	25.8 (22.3–29.4)	22.7 (19.1–26.3)	14.3 (11.3–17.2)	8.2 (6.0–10.5)
	R	1.8 (1.5–2.2)	1.6 (1.3–2.0)	1.4 (1.1–1.7)	1.1 (0.8–1.3)	1.0 (0.7–1.2)
	PAF (%)	58.3 (57.6–59.1)	50.2 (49.6–50.9)	21.1 (20.8–21.5)	9.7 (9.4–10.0)	5.1 (4.9–5.3)
	PAR	19.6 (17.3–21.9)	16.0 (14.0–18.0)	6.0 (5.0–7.0)	2.2 (1.5–2.9)	1.0 (0.5–1.5)

WHO HEAT; indicator MNCH7; Ghana DHS/MICS. D, Difference (percentage points); R, Ratio; PAF, Population Attributable Fraction (%); PAR, Population Attributable Risk (percentage points). 95% CIs in parentheses. Full results for all nine survey years are provided in Supplementary file 1.

Educational inequality similarly narrowed: D decreased from 53.6 (95% CI: 47.2–59.9) in 1993 to 17.8 (95% CI: 13.7–21.9) in 2022, and PAF fell from 89.2% to 12.5%. Rural–urban inequality (D) declined from 35.8 (95% CI: 30.5–41.0) to 9.7 (95% CI: 7.2–12.2) over the same period. Regional inequality (D) fell from 28.1 (95% CI: 24.0–32.1) in 1993 to 8.2 (95% CI: 6.0–10.5) in 2022. Age-related inequality remained negligible across all survey years, with D consistently below 0.3 percentage points and R at approximately 1.0 (Supplementary file 1).

## Discussion

This study aimed to explore the trends and inequalities in health facility deliveries among women of reproductive age in Ghana from 1993 to 2022. The results show a significant rise in institutional deliveries nationwide, from 42.3% in 1993 to 85.4% in 2022. The observed increase in facility deliveries coincides temporally with the introduction of the Free Maternal Health Policy (2008) and the expansion of the National Health Insurance Scheme (2005). However, this descriptive analysis cannot establish causality (4, 26). Furthermore, the increase in facility-based deliveries aligns with global trends that have focused on improving access to institutional maternal care (27).

Despite the overall national progress, considerable disparities remain, particularly in relation to regional, educational, and economic factors. For example, the Upper East region had the highest prevalence of facility-based deliveries at 97.7%, with other regions such as Greater Accra and Ashanti showing similar improvements. The observed improvements coincided with policy reforms such as the Free Maternal Health Policy and NHIS; however, this descriptive analysis cannot establish causality. This is in line with earlier studies that report significant geographic disparities in healthcare access across countries (28–30). This highlights the need for targeted investments and healthcare initiatives aimed at underserved regions to ensure equitable access to maternal healthcare services.

The study also reveals that education and economic status are major determinants of health facility delivery rates. Women with higher education levels consistently had higher facility-based delivery rates, reaching nearly 98% by 2022, while women with no formal education had significantly lower rates, at 72.3%. These findings are consistent with global research showing that higher education is strongly associated with greater healthcare access and utilization (17, 22). In addition, women from wealthier backgrounds were more likely to deliver in health facilities compared to those from poorer backgrounds, underscoring the pro-rich inequality in healthcare access. Although the poorest quintile saw significant improvements, their coverage only reached 71.1% by 2022, indicating that economic barriers to access remain a challenge. This observation aligns with studies from Ghana and other low- and middle-income countries, where wealth remains a significant barrier to healthcare access (31–33).

The urban-rural divide remains a key factor in access to health facility deliveries. Urban populations consistently had higher delivery rates in health facilities compared to their rural counterparts. While rural areas showed substantial improvements over the study period, they continued to lag behind urban areas. This reflects long-standing disparities in infrastructure, healthcare provider availability, and access to transportation, which are more accessible in urban settings (34). These regional disparities point to the need for focused efforts to enhance healthcare infrastructure and services in rural areas, ensuring that all women, regardless of their location, have access to skilled birth attendance. The findings also showed that age and economic status disparities remained largely stable over time. Women in the 20–49 age group continued to have higher rates of facility-based deliveries compared to those aged 15–19, although the gap between the two groups remained narrow. This trend suggests that improvements in healthcare access benefit women across different age groups, although age-related disparities still exist in other areas of maternal health, such as antenatal care and postnatal services (35, 36). Moreover, the reduction in the D statistic and PAF values indicates that economic inequalities in facility delivery rates have decreased over time, though significant gaps remain. These trends mirror global patterns, where economic inequalities persist even as overall healthcare access improves (37). While significant progress has been made in improving maternal healthcare access in Ghana, inequalities related to education, economic status, and region continue to affect the equitable distribution of health services. These findings highlight the importance of focusing on equitable access to healthcare, particularly for rural, economically disadvantaged, and less educated populations.

### Implications for policy, research, and practice

The findings highlight persistent subnational disparities that warrant targeted policy attention. Regions with persistently lower facility delivery rates, particularly in northern Ghana, may benefit from focused investments in healthcare infrastructure, transportation networks, and healthcare worker deployment. Addressing financial barriers through mechanisms such as conditional cash transfers or expanded coverage under the NHIS could reduce economic inequalities in facility delivery access. Community-based demand-generation activities and awareness programmes, particularly in areas with low educational attainment, may help shift health-seeking behaviour towards facility-based delivery.

Further research is needed to understand the mechanisms underlying persistent regional and socioeconomic disparities. Qualitative studies exploring the cultural, logistical, and economic barriers experienced by women in underserved regions would complement the trend data presented here. Longitudinal evaluations that prospectively assess the impact of specific policy interventions, rather than relying on descriptive trend analysis, are needed to establish which strategies are most effective in reducing inequality. Additionally, research on the role of community health workers and community-based interventions in improving facility delivery coverage in remote areas would provide valuable evidence for programme design.

For healthcare practitioners, the findings reinforce the importance of reaching women in rural and economically disadvantaged communities. Expanding mobile health services, establishing community-based health posts, and ensuring that health workers in rural areas receive appropriate training and support are practical measures that could help address the remaining rural–urban gap. Healthcare providers should also engage in educational outreach about the importance of facility-based delivery, particularly in areas with lower literacy rates and greater reliance on traditional birth practices.

### Strengths and limitations

This study has several strengths. The longitudinal perspective spanning nearly three decades (1993–2022) provides a comprehensive picture of progress and persistent inequalities in health facility deliveries in Ghana. The use of WHO HEAT ensures consistent, age-standardised, and weighted estimates across nine survey rounds, enabling valid trend comparisons. The analysis examines multiple dimensions of inequality using four complementary summary measures (D, R, PAF, PAR), providing a nuanced characterisation of inequalities.

However, several limitations should be noted. This analysis uses aggregated secondary data; individual-level analysis was not possible, and no causal inference can be drawn from descriptive time-trend data alone. The inequality measures are bivariate and do not adjust for confounding between dimensions. DHS/MICS questionnaire wording and recall periods have changed slightly over time, which may affect comparability across survey rounds. Changes in Ghana's regional boundaries (e.g., the creation of six new regions in 2018) were harmonised by HEAT, but some historical regional comparisons may nonetheless be affected. Sample sizes for smaller regions (e.g., Oti, Savannah) are limited, leading to wide confidence intervals in those areas. Finally, this analysis cannot explain why inequalities persist; qualitative research is needed to explore the underlying social, cultural, infrastructural, and system-level barriers.

## Conclusions

Health facility delivery prevalence in Ghana rose markedly from 42.3% in 1993 to 85.4% in 2022, with absolute inequalities narrowing across all dimensions examined. Nonetheless, in 2022, marked disparities persisted by wealth, education, place of residence, and region. These observed trends coincide temporally with policy reforms including the Free Maternal Health Policy and the National Health Insurance Scheme; however, this descriptive analysis cannot establish causality. Achieving equitable access to facility-based delivery will require continued and targeted investments in healthcare infrastructure, financial protection mechanisms, and demand-generation strategies, particularly for women in rural areas, in the lowest wealth quintiles, and with little or no formal education.

## Data Availability

Publicly available datasets were analyzed in this study. This data can be found here: https://www.who.int/data/inequality-monitor/assessment_toolkit.
