# Supplementary material for: Trends and inequalities in health facility deliveries among women of reproductive age in Ghana, 1993–2022
Source: Front Reprod Health. 2026 Jul 3;8:1874042. doi: 10.3389/frph.2026.1874042 (PMC13376258; doi:10.3389/frph.2026.1874042)
Supplement: Supplementary file 1 [file Table1.docx]

**Table 1: Summary measures of inequalities with deliveries in health facilities in Ghana by different inequality dimensions, 1993-2022**

|  |  | **1993** | | | **1998** | | | **2003** | | | **2006** | | | **2008** | | | **2011** | | | **2014** | | | **2017** | | | **2022** | | |
| --- | --- | --- | --- | --- | --- | --- | --- | --- | --- | --- | --- | --- | --- | --- | --- | --- | --- | --- | --- | --- | --- | --- | --- | --- | --- | --- | --- | --- |
| **Dimension** | **Measure** | **Est. (%)** | **CI-LB** | **CI-UB** | **Est. (%)** | **CI-LB** | **CI-UB** | **Est. (%)** | **CI-LB** | **CI-UB** | **Est. (%)** | **CI-LB** | **CI-UB** | **Est. (%)** | **CI-LB** | **CI-UB** | **Est. (%)** | **CI-LB** | **CI-UB** | **Est. (%)** | **CI-LB** | **CI-UB** | **Est. (%)** | **CI-LB** | **CI-UB** | **Est. (%)** | **CI-LB** | **CI-UB** |
| Economic status (wealth quintile) | D | 60.62 | 53.51 | 67.73 | 65.05 | 59.44 | 70.66 | 70.06 | 65.15 | 74.97 | 68.16 | 61.04 | 75.28 | 69.22 | 64.34 | 74.11 | 59.66 | 54.83 | 64.48 | 50.59 | 44.02 | 57.16 | 34.52 | 28.16 | 40.88 | 25.73 | 19.55 | 31.91 |
|  | PAF | 98.96 | 98.85 | 99.07 | 96.24 | 96.15 | 96.33 | 95.95 | 95.87 | 96.03 | 83.99 | 83.88 | 84.11 | 62.39 | 62.33 | 62.45 | 44.59 | 44.54 | 44.63 | 32.15 | 32.13 | 32.17 | 24.13 | 24.11 | 24.16 | 13.30 | 13.29 | 13.32 |
|  | PAR | 41.86 | 37.21 | 46.51 | 41.95 | 37.88 | 46.03 | 43.80 | 40.23 | 47.37 | 40.91 | 35.24 | 46.57 | 35.63 | 32.19 | 39.08 | 30.04 | 27.22 | 32.86 | 23.50 | 21.78 | 25.21 | 18.80 | 16.82 | 20.77 | 11.37 | 9.94 | 12.79 |
|  | R | 3.58 | 2.80 | 4.57 | 4.17 | 3.47 | 5.02 | 4.61 | 3.83 | 5.57 | 4.18 | 3.25 | 5.36 | 3.94 | 3.35 | 4.64 | 2.58 | 2.30 | 2.90 | 2.10 | 1.83 | 2.41 | 1.56 | 1.41 | 1.72 | 1.36 | 1.25 | 1.48 |
| Age (15–49) | D | 0.2 | -4.3 | 4.6 | 0.1 | -4.2 | 4.4 | 0.3 | -4.2 | 4.8 | 0.0 | -4.1 | 4.3 | 0.0 | -4.2 | 4.2 | 0.1 | -4.2 | 4.5 | 0.1 | -4.1 | 4.3 | 0.2 | -4.2 | 4.6 | 0.2 | -4.1 | 4.5 |
|  | PAF | 0.3 | -6.0 | 6.5 | 0.2 | -5.7 | 6.2 | 0.4 | -6.2 | 7.0 | 0.0 | -5.9 | 6.0 | 0.0 | -6.0 | 6.0 | 0.2 | -6.0 | 6.4 | 0.1 | -6.0 | 6.1 | 0.2 | -6.1 | 6.5 | 0.3 | -5.9 | 6.4 |
|  | PAR | 0.1 | -2.1 | 2.2 | 0.1 | -2.0 | 2.1 | 0.1 | -2.1 | 2.3 | 0.0 | -2.0 | 2.1 | 0.0 | -2.0 | 2.0 | 0.1 | -2.1 | 2.2 | 0.0 | -2.0 | 2.1 | 0.1 | -2.1 | 2.2 | 0.1 | -2.0 | 2.1 |
|  | R | 1.0 | 0.9 | 1.1 | 1.0 | 0.9 | 1.1 | 1.0 | 0.9 | 1.1 | 1.0 | 0.9 | 1.1 | 1.0 | 0.9 | 1.1 | 1.0 | 0.9 | 1.1 | 1.0 | 0.9 | 1.1 | 1.0 | 0.9 | 1.1 | 1.0 | 0.9 | 1.1 |
| Education (4 groups) | D | 53.6 | 47.2 | 59.9 | 55.4 | 50.0 | 60.9 | 58.7 | 53.9 | 63.5 | 55.8 | 49.2 | 62.4 | 50.9 | 45.8 | 56.0 | 46.2 | 41.7 | 50.7 | 41.6 | 36.3 | 46.8 | 28.7 | 23.6 | 33.8 | 17.8 | 13.7 | 21.9 |
|  | PAF | 89.2 | 88.7 | 89.8 | 85.7 | 85.3 | 86.2 | 80.9 | 80.4 | 81.4 | 72.5 | 72.0 | 73.0 | 55.8 | 55.4 | 56.2 | 41.6 | 41.3 | 41.9 | 30.3 | 30.0 | 30.6 | 21.9 | 21.7 | 22.2 | 12.5 | 12.3 | 12.7 |
|  | PAR | 28.3 | 25.6 | 31.0 | 28.5 | 25.9 | 31.1 | 26.7 | 24.6 | 28.8 | 23.3 | 20.5 | 26.2 | 18.4 | 16.6 | 20.3 | 14.2 | 12.8 | 15.7 | 10.5 | 9.0 | 12.0 | 6.9 | 5.6 | 8.3 | 3.3 | 2.5 | 4.2 |
|  | R | 3.2 | 2.6 | 4.0 | 3.3 | 2.8 | 4.0 | 3.5 | 2.9 | 4.2 | 3.0 | 2.5 | 3.6 | 2.6 | 2.2 | 3.1 | 2.0 | 1.7 | 2.3 | 1.7 | 1.4 | 2.0 | 1.3 | 1.1 | 1.5 | 1.1 | 0.9 | 1.3 |
| Residence (urban/rural) | D | 35.8 | 30.5 | 41.0 | 38.9 | 34.2 | 43.7 | 39.5 | 35.1 | 43.9 | 36.7 | 31.0 | 42.4 | 33.3 | 28.6 | 38.0 | 28.4 | 24.4 | 32.4 | 23.1 | 19.2 | 27.0 | 16.9 | 13.8 | 20.0 | 9.7 | 7.2 | 12.2 |
|  | PAF | 65.0 | 64.1 | 65.9 | 59.5 | 58.7 | 60.3 | 54.1 | 53.3 | 54.9 | 45.7 | 44.9 | 46.5 | 33.7 | 33.1 | 34.3 | 23.7 | 23.2 | 24.2 | 16.2 | 15.8 | 16.6 | 11.2 | 10.9 | 11.5 | 6.1 | 5.9 | 6.3 |
|  | PAR | 23.6 | 20.6 | 26.6 | 22.9 | 20.1 | 25.7 | 21.6 | 19.1 | 24.1 | 18.1 | 15.1 | 21.1 | 13.9 | 11.9 | 15.9 | 9.6 | 8.0 | 11.3 | 6.2 | 4.9 | 7.6 | 4.0 | 2.9 | 5.2 | 1.8 | 1.1 | 2.6 |
|  | R | 2.1 | 1.7 | 2.6 | 2.4 | 2.0 | 2.9 | 2.5 | 2.0 | 3.0 | 2.1 | 1.7 | 2.6 | 1.9 | 1.6 | 2.3 | 1.6 | 1.3 | 1.9 | 1.5 | 1.2 | 1.8 | 1.2 | 1.0 | 1.5 | 1.1 | 0.9 | 1.3 |
| Subnational region | D | 28.1 | 24.0 | 32.1 | 27.7 | 23.8 | 31.6 | 25.8 | 22.3 | 29.4 | 24.5 | 20.5 | 28.6 | 23.9 | 20.2 | 27.6 | 22.7 | 19.1 | 26.3 | 19.6 | 16.1 | 23.1 | 14.3 | 11.3 | 17.2 | 8.2 | 6.0 | 10.5 |
|  | PAF | 58.3 | 57.6 | 59.1 | 54.9 | 54.3 | 55.6 | 50.2 | 49.6 | 50.9 | 43.8 | 43.2 | 44.4 | 31.6 | 31.2 | 32.0 | 21.1 | 20.8 | 21.5 | 14.3 | 14.0 | 14.7 | 9.7 | 9.4 | 10.0 | 5.1 | 4.9 | 5.3 |
|  | PAR | 19.6 | 17.3 | 21.9 | 18.3 | 16.1 | 20.6 | 16.0 | 14.0 | 18.0 | 12.9 | 10.8 | 15.0 | 9.6 | 8.2 | 11.0 | 6.0 | 5.0 | 7.0 | 3.8 | 3.0 | 4.6 | 2.2 | 1.5 | 2.9 | 1.0 | 0.5 | 1.5 |
|  | R | 1.8 | 1.5 | 2.2 | 1.7 | 1.4 | 2.1 | 1.6 | 1.3 | 2.0 | 1.5 | 1.2 | 1.9 | 1.5 | 1.2 | 1.8 | 1.4 | 1.1 | 1.7 | 1.3 | 1.0 | 1.6 | 1.1 | 0.8 | 1.3 | 1.0 | 0.7 | 1.2 |
